# Differences in choroidal responses to near work between myopic children and young adults

**DOI:** 10.1186/s40662-024-00382-5

**Published:** 2024-04-02

**Authors:** Mengqi Liu, Yuanyuan Wang, Haoer Li, Yunpeng Zhao, Min Ma, Shihan Xu, Xiaohuan Wei, Ruiyan Xu, Ruikang Tian, Xiangtian Zhou, Hao Wu

**Affiliations:** 1 National Clinical Research Center for Ocular Diseases, Eye Hospital, Wenzhou Medical University, Wenzhou, 325027 China; 2 State Key Laboratory of Ophthalmology, Optometry and Visual Science, Eye Hospital, Wenzhou Medical University, Wenzhou, 325027 China; 3 Research Unit of Myopia Basic Research and Clinical Prevention and Control, Chinese Academy of Medical Sciences (2019RU025), Wenzhou, Zhejiang China

**Keywords:** Choroidal vascularity, Choriocapillaris, Near work, Myopia susceptibility

## Abstract

**Background:**

Near work is generally considered as a risk factor for myopia onset and progression. This study aimed to investigate the choroidal responses to a brief-period of near work in children and young adults.

**Methods:**

Thirty myopic medical students (aged 18–28 years) and 30 myopic children (aged 8–12 years) participated in this study. The submacular total choroidal area (TCA), luminal area (LA), stromal area (SA), choroidal vascularity index (CVI) and choriocapillaris flow deficit (CcFD), as well as subfoveal choroidal thickness (SFCT) were measured with swept-source optical coherence tomography/optical coherence tomography angiography (SS-OCT/OCTA) before and immediately after 20 min, 40 min, 60 min of near work at a distance of 33 cm.

**Results:**

In adults, 20 min of near work induced a significant reduction in SFCT (− 5.1 ± 6.5 μm), LA [(− 19.2 ± 18.6) × 10^3^ μm^2^], SA [(− 8.2 ± 12.6) × 10^3^ μm^2^] and TCA [(− 27.4 ± 24.9) × 10^3^ μm^2^] (all *P* < 0.01). After 40 min of near work, LA was still reduced [(− 9.4 ± 18.3) × 10^3^ μm^2^], accompanied with a decreased CVI (− 0.39% ± 0.70%) and an increased CcFD (0.30% ± 0.78%) (all *P* < 0.05). After 60 min of near work, CVI was still reduced (− 0.28% ± 0.59%), and CcFD was still increased (0.37% ± 0.75%) (all *P* < 0.05). In children, 20 min of near work induced a significant increase in CcFD (0.55% ± 0.64%), while 60 min of near work induced increases in SA [(7.2 ± 13.0) × 10^3^ μm^2^] and TCA [(9.7 ± 25.3) × 10^3^ μm^2^] and a reduction in CVI (− 0.28% ± 0.72%) (all *P* < 0.05). Children exhibited lower near work-induced LA and TCA reduction than adults, with a mean difference of − 0.86% and − 0.82%, respectively (all *P* < 0.05).

**Conclusions:**

The temporal characteristics and magnitude of changes of choroidal vascularity and choriocapillaris perfusion during near work was not identical between children and adults. The initial response to near work was observed in choriocapillaris in children, whereas it was observed in the medium- and large-sized vessels in adults.

*Trial registration*: Clinical Trial Registry (ChiCTR), ChiCTR2000040205. Registered on 25 November 2020, https://www.chictr.org.cn/bin/project/edit?pid=64501.

**Supplementary Information:**

The online version contains supplementary material available at 10.1186/s40662-024-00382-5.

## Background

Near work is generally thought to be a potential influencer for myopia onset and progression in cross-sectional and longitudinal studies [1]. Even though the burden of near work is not reduced in young adults, such as medical students, the rate of myopia progression is not as rapid as that in schoolchildren [2–6]. Such age-related difference in susceptibility to myopia has been demonstrated in animal models of experimentally induced myopia as well [7, 8]. However, the reason behind such differences is unknown.

The choroid is a highly vascularized tissue, exhibiting developmental changes during childhood and adolescence. Choroidal thickness tends to increase in early childhood [9–11], then reaches a peak in young adulthood, followed by a reduction with age in older individuals [12, 13]. Choroidal vascularity index (CVI) decreases with age in children and adults [14, 15], while choriocapillaris flow deficit percentage (CcFD%) was reported to be higher in adults than in children [14, 16]. Such developmental changes in choroidal vasculature suggest that the choroidal responses to near work might vary across these age cohorts.

Increasing evidence implicate the choroid to be involved in ocular growth regulation and myopia development through vision-driven local retina-choroid-scleral molecular signaling cascades [17, 18]. Recent findings from a near work guinea pig model showed that myopia development was accompanied by decreases in choroidal thickness and choroidal perfusion [19]. Clinical studies in children and young adults also reported choroidal thinning and reduction in choroidal perfusion in response to near work [20–25]. Nevertheless, it remains unknown whether these choroidal responses differ between children and young adults.

To this end, we here investigated the choroidal vascularity and choriocapillaris perfusion in response to near work in children and adults (medical students) and compared the temporal choroidal characteristics and magnitude of choroidal changes between these cohorts. The findings of this study will hopefully provide new insights for future studies exploring the role of the choroid in relation to near work and myopia development in different age groups.

## Methods

### Participants

Thirty medical students (aged 18–28 years) from Wenzhou Medical University and 30 children (aged 8–12 years) from the two prospective study cohorts and optometry outpatients at the Eye Hospital of Wenzhou Medical University were invited to participate in this study between January 2021 and May 2022. All participants were in good health and met the following inclusion criteria: (1) spherical equivalent refraction (SER) between − 6.00 diopters (D) and − 0.50 D; (2) astigmatism of less than 1.00 D; (3) best corrected visual acuity (BCVA) of less than 0.0 logMAR; (4) intraocular pressure (IOP) of less than 21 mmHg; (5) amplitude of accommodation ≥ 6.00 D. Strabismus or amblyopia, smoking, previous eye surgery or trauma, other chronic ocular diseases and systemic diseases were the exclusion criteria. Subjects undergoing myopia control treatments (such as atropine drops, orthokeratology lenses, etc.) and use of prescription medicines were also excluded. All participants and the parents of children signed an informed consent. The study was approved by the ethics committee of the Eye Hospital, Wenzhou Medical University (2020-173-K-158-01).

### Measurements and procedures

Medical histories were collected, and ophthalmologic examinations were performed to ascertain whether the subjects met the criteria. Non-cycloplegic subjective refraction was measured using a phoropter (RT-5100, NIDEK CO., LTD., Japan). IOP was measured by non-contact tonometry (Canon TX-20, Canon Inc., Japan). Axial length (AL), and corneal refractive power were measured using an IOL Master 700 (Carl Zeiss Meditec AG, Germany). The amplitude of accommodation was measured by the push-up method using a Royal Air Force binocular gauge (Haag-Streit England, United Kingdom).

The participants were required to refrain from alcohol and caffeine for at least 24 h before each visit. The examinations were carried out at the same time (01:30 p.m. to 05:30 p.m.) over 3 separate days to minimize the influence of diurnal rhythms. In order to abrogate the effect of any visual tasks the subjects may have carried out before the visit, they initially watched a movie on a 65-inch television (65A57F, Hisense, China) at a distance of 3 meters for 15 min with their refractive errors fully corrected with trial lenses. Then, choroidal images of the right eyes were captured with SS-OCT/OCTA (VG200S, SVision Imaging, China) at baseline. This was followed by a near work activity, which in this case was to read an e-book on an Android tablet (HUAWEI AGS2-W09, 10.1 inches, screen resolution 1920 × 1200, screen luminance 200 nits) at a distance of 33 cm (3.00 D accommodation demand) for either 20, 40 or 60 min. The sequence of the three visits was randomized among subjects (Additional file 1). The participants read books they were interested in under the supervision of an investigator to ensure they were not distracted. The investigator recorded the page number before and after the reading task and checked the viewing distance every 5 min. In each visit, participants were asked to summarize what they had read to ensure they were actively reading. The texts were set in the same font, size, and line spacing (Chinese, 10.5 pt in Microsoft YaHei and double spaced) with dark letters on a bright background. After completion of the reading task, participants moved to the SS-OCT/OCTA and choroidal images were captured. The room light was maintained at 200 to 300 lx.

### SS-OCT/OCTA imaging and analysis

The SS-OCT/OCTA system contains a swept-source laser with a central wavelength of approximately 1050 nm and a scan rate of 200,000 A-scans per second. The axial resolution, lateral resolution, and scan depth were 5 µm, 13 µm, and 3 mm, respectively.

**Fig. 1:**
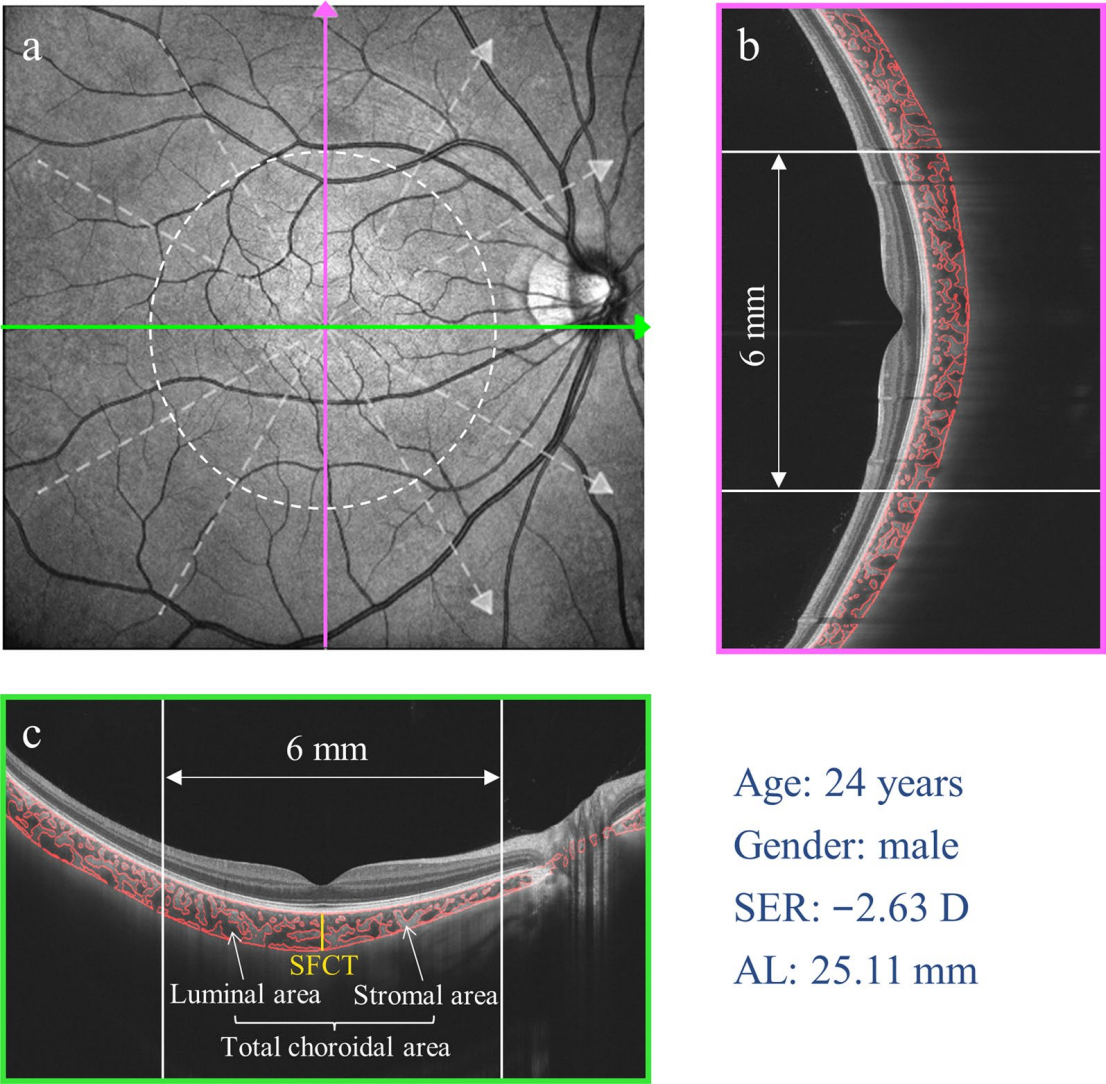
Illustration of choroidal vasculature analysis. **a** Schematic diagram of structural optical coherence tomography (OCT) imaging of the macular region with 6 radial scan lines centered on the fovea. **b**, **c** Overlays of binarized choroidal areas on original images in vertical (**b**) and horizontal (**c**) meridian. The 6-mm wide submacular area between white vertical lines, centered on the fovea, was used for analysis. The yellow vertical line represented the subfoveal choroidal thickness (SFCT). SER,  spherical equivalent refraction; AL,  axial length

Structural OCT imaging of the macular region was performed with 6 radial scan lines centered on the fovea (Fig. 1). Each scan line, generated by 2048 A-scans, was 12 mm long and separated from adjacent lines by 30 degrees. Sixty-four B-scans were obtained on each scan line and were automatically averaged to improve the signal-to-noise ratio. The images were analyzed with a fully automatic method. The segmentation of the choroidal boundaries was based on deep learning algorithms, ResNet-UNet neural network [26, 27]. Binarization was performed for the choroidal images based on Niblack’s auto local threshold, using custom-designed algorithms in MATLAB R2017a (MathWorks, Natick, MA, USA) [26, 27]. The vessels and stroma in the choroid were demarcated with red dotted lines. The submacular total choroidal area (TCA), luminal area (LA), and stromal area (SA) in a 6 mm submacular region centered on the fovea, as well as subfoveal choroidal thickness (SFCT) were calculated. The CVI was defined as the ratio of LA to TCA [28, 29]. All choroidal metrics were estimated by calculating the mean values of the vertical and horizontal meridians (Fig. 1).

**Fig. 2:**
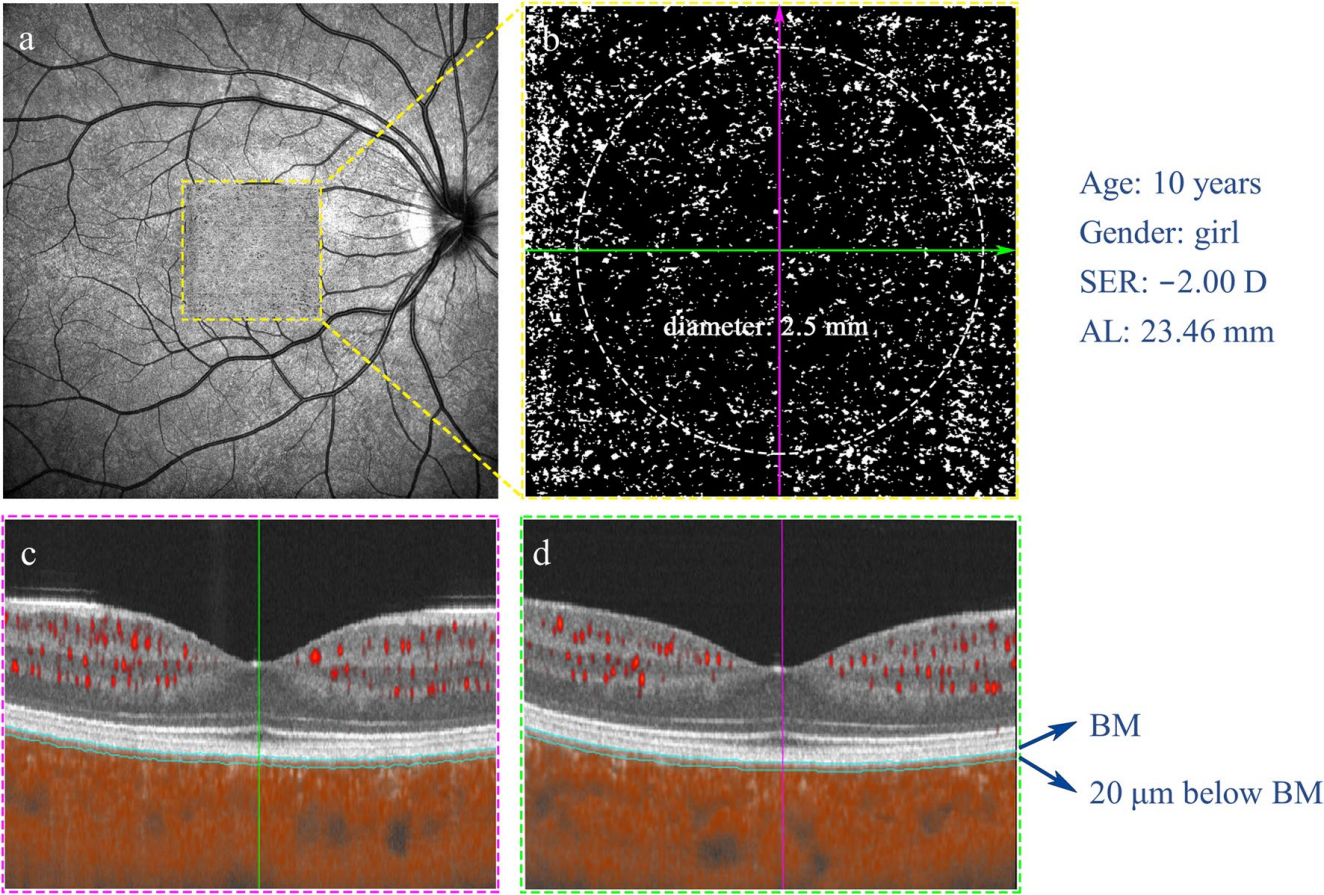
Illustration of choriocapillaris perfusion analysis. **a** Optical coherence tomography angiography (OCTA) scan region of nominally 3 mm × 3 mm, centered on the fovea. **b** Magnified *en face* OCTA choriocapillaris image within a 2.5 mm diameter circle, which was centered on the fovea through examining (**c**) vertical and (**d**) horizontal meridian scans across the fovea and was segmented as a 20 µm thickness slab below Bruch’s membrane (BM). SER,  spherical equivalent refraction; AL,  axial length

OCTA fundus images were obtained with a raster scan protocol of 512 horizontal B-scans that covered an area of 3 mm × 3 mm centered on the fovea (Fig. 2). The B-scans, which contained 512 A-scans each, were repeated 6 times and averaged. The choriocapillaris layer was defined as a slab from the basal border of the retinal pigment epithelium (RPE)-Bruch’s membrane (BM) complex to 20 µm below it [30], which was recognized by the machine-built algorithm automatically and corrected manually if necessary. Each image was compensated to remove retinal vessel projection artifacts and to adjust for shadowing artifacts [31]. The choriocapillaris flow deficit (CcFD), indicating the region with absence of flow signals from choriocapillaris, was analyzed with machine-built algorithms [a global thresholding method with a threshold of 1.5 times the standard deviation (1.5 × SD method)] [31]. This method utilizes a mean SD of a normal database that included 19 subjects aged between 20 and 35 years, which was approximately 30 in gray level, and the binarized image of CcFD closely resembles that in the original OCTA image [30]. The CcFD was calculated in a 2.5 mm diameter circular region centered on the fovea by examining the B-scans vertically and horizontally (Fig. 2).

An OCT and OCTA signal strength of above 8 was deemed fit for further analysis. The scale of both OCT and OCTA images were adjusted for the differences in magnification due to differences in ocular length [32]. All images were acquired using the follow-up mode in the equipment that ensured the same choroidal region was captured on each visit. Repeatability was assessed by capturing two sets of images at the first visit. The intraclass correlation coefficients (ICCs) and Bland–Altman plots showed excellent repeatability between the two series of images (Additional file 2). Meanwhile, the repeatability from session to session was high, with ICCs varying from 0.918 to 0.992 for the pre-near work choroidal metrics. Additionally, no significant differences in pre-near work choroidal metrics were observed in both adults and children during the three visits (all *P* > 0.05, Additional files 3 and 4).

### Statistical analysis

Statistical analysis was performed using IBM SPSS Statistics (version 27, IBM, NY, USA). The results are presented as mean ± SD wherever applicable. The Shapiro–Wilk's test was used to confirm that the data sets were normally distributed. Two-way repeated measures ANOVA was used to analyze the effect of near work on choroidal metrics at different time points. When the assumption of sphericity was violated, the *P* value was adjusted with the Greenhouse–Geisser. Bonferroni adjustment was applied for pairwise comparison tests. Generalized estimating equations (GEE) was used to compare the magnitude of changes between the groups and time, adjusted for AL. *P* < 0.05 was defined as statistically significant.

## Results

### Demographics and ocular metrics of the studied participants

Thirty-one adults and 32 children participated in the study. One adult and two children dropped out of the study midway. Finally, 30 adults (12 males and 18 females) and 30 children (13 boys and 17 girls) with a mean age of 23.3 ± 2.3 years and 9.9 ± 1.0 years, respectively, were included in the analyses. The mean SER and AL of adults were − 3.50 ± 0.95 D (range: − 5.37 to − 1.62 D) and 25.00 ± 1.05 mm (range: 23.27 to 27.16 mm), respectively, while the mean SER and AL of children were − 2.00 ± 0.91 D (range: − 3.87 to − 0.50 D) and 24.56 ± 0.67 mm (range: 23.46 to 25.87 mm), respectively (Table 1). The corneal refractive power of adults and children were 43.54 ± 1.53 D (range: 39.98 to 46.21 D) and 43.11 ± 1.53 D (range: 40.27 to 46.42 D), respectively. The IOP of adults and children were 14.1 ± 2.7 mmHg (range: 8.1 to 20.1 mmHg) and 14.7 ± 2.7 mmHg (range: 10.0 to 19.3 mmHg), respectively. The mean amplitude of accommodation of adults was 11.0 ± 2.0 D (range: 7.0 to 15.0 D), which was 13.7 ± 2.5 D (range: 8.5 to 19.3 D) in children. The children showed higher SER and amplitude of accommodation (all *P* < 0.001), while other parameters were not significantly different between the two groups (all *P* > 0.05).

**Table 1:** Demographics and ocular metrics of the study population

Parameter	Adults (n = 30)		Children (n = 30)		*P* value
	Mean ± SD	Range	Mean ± SD	Range	
Age (years)	23.3 ± 2.3	18.0–28.0	9.9 ± 1.0	9.0–12.0	< 0.001^‡^
Sex (male:female)	12:18		13:17		0.793^*^
Dominant eye (OD:OS)	18:12		20:10		0.592^*^
SER (D)	− 3.50 ± 0.95	− 5.37 to − 1.62	− 2.00 ± 0.91	− 3.87 to − 0.50	< 0.001^†^
AL (mm)	25.00 ± 1.05	23.27–27.16	24.56 ± 0.67	23.46–25.87	0.054^†^
CP (D)	43.54 ± 1.53	39.98–46.21	43.11 ± 1.53	40.27–46.42	0.276^†^
IOP (mmHg)	14.1 ± 2.7	8.1–20.1	14.7 ± 2.7	10.0–19.3	0.472^†^
Amp of Acc (D)	11.0 ± 2.0	7.0–15.0	13.7 ± 2.5	8.5–19.3	< 0.001^†^
SFCT (μm)	267.2 ± 68.6	149.8–419.4	255.9 ± 52.8	152.2–348.5	0.475^†^
LA (× 10^3^μm^2^)	990.0 ± 229.3	569.3–1358.9	919.6 ± 167.6	602.3–1201.3	0.180^†^
SA (× 10^3^μm^2^)	589.4 ± 113.7	370.5–785.9	587.8 ± 115.4	318.8–801.6	0.956^†^
TCA (× 10^3^μm^2^)	1579.4 ± 331.8	986.7–2119.1	1507.4 ± 269.6	921.0–1970.0	0.360^†^
CVI (%)	62.45 ± 3.08	56.57–67.36	61.06 ± 2.90	53.74–65.34	0.078^†^
CcFD (%)	8.41 ± 2.20	5.98–13.87	7.05 ± 1.33	4.79–9.73	0.019^‡^

*SER* = spherical equivalent refraction; *AL* = axial length; *CP* = corneal refractive power; *IOP* = intraocular pressure; *Amp of Acc* = amplitude of accommodation; *SFCT* = subfoveal choroidal thickness; *LA* = luminal area; *SA* = stromal area; *TCA* = total choroidal area; *CVI* = choroidal vascularity index; *CcFD* = choriocapillaris flow deficits; *SD* = standard deviation

^†^*P* value determined by independent t-test

^‡^*P* value determined by Mann–Whitney U test

^*^P value determined by Pearson chi-squared test

The baseline SFCT was 267.2 ± 68.6 μm in adults, and it was 255.9 ± 52.8 μm in children (Table 1). The submacular LA, SA and TCA in adults were (990.0 ± 229.3) × 10^3^ μm^2^, (589.4 ± 113.7) × 10^3^ μm^2^ and (1579.4 ± 331.8) × 10^3^ μm^2^, respectively. The LA, SA and TCA in children were (919.6 ± 167.6) × 10^3^ μm^2^, (587.8 ± 115.4) × 10^3^ μm^2^, and (1507.4 ± 269.6) × 10^3^ μm^2^, respectively. The CVI in adults was 62.45% ± 3.08%, which was 61.06% ± 2.90% in children. All the above parameters were not significantly different between the two groups. Only the CcFD was higher in adults than in children (8.41% ± 2.20% *vs.* 7.05% ± 1.33%, *P* = 0.019).

### Influence of near work on choroidal metrics in adults

Near work for 20 min induced a significant decrease in SFCT (− 5.1 ± 6.5 μm, *P* < 0.001), as well as decreases in LA [(− 19.2 ± 18.6) × 10^3^ μm^2^, *P* < 0.001], SA [(− 8.2 ± 12.6) × 10^3^ μm^2^, *P* = 0.001] and TCA [(− 27.4 ± 24.9) × 10^3^ μm^2^, *P* < 0.001], whereas no significant changes were observed for CVI and CcFD (Table 2, Additional file 3). After 40 min of near work, LA still declined [(− 9.4 ± 18.3) × 10^3^ μm^2^, *P* = 0.009], as well as CVI (− 0.39% ± 0.70%, *P* = 0.005), and CcFD increased (0.30% ± 0.78%, *P* = 0.045). A significant decrease in CVI and increase in CcFD was also observed after 60 min of near work (CVI: − 0.28% ± 0.59%, *P* = 0.015; CcFD: 0.37% ± 0.75%, *P* = 0.012).

**Table 2:** Trend of choroidal metrics after near work

Trend	Adults			Children		
	20 min	40 min	60 min	20 min	40 min	60 min
SFCT	↓^***^					
LA	↓^***^	↓^**^				
SA	↓^**^					↑^**^
TCA	↓^***^					↑^*^
CVI		↓^**^	↓^*^			↓^*^
CcFD		↑^*^	↑^*^	↑^***^		

*SFCT* = subfoveal choroidal thickness; *LA* = luminal area; *SA* = stromal area; *TCA* = total choroidal area; *CVI* = choroidal vascularity index; *CcFD* = choriocapillaris flow deficits

^*^P < 0.05; **P < 0.01; ***P < 0.001

**↓**Near work decreases the parameter; **↑** Near work increases the parameter

### Influence of near work on choroidal metrics in children

Compared to pre-near work values, CcFD increased significantly after 20 min of near work (0.55% ± 0.64%, *P* < 0.001), with no significant changes in SFCT, LA, SA, TCA and CVI (all *P* > 0.05, Table 2, Additional file 4). After 40 min of near work, we did not witness the above choroidal metrics changing significantly. After 60 min of near work, SA increased [(7.2 ± 13.0) × 10^3^ μm^2^, *P* = 0.005], accompanied by an increase in TCA [(9.7 ± 25.3) × 10^3^ μm^2^, *P* = 0.046] and a decrease in CVI (− 0.28% ± 0.72%, *P* = 0.040).

### Differences in choroidal changes during near work between children and adults

The comparison of choroidal changes during near work were adjusted for AL and these changes were expressed as percentages, which were defined as the ratio of choroidal metrics at post-near work minus those at pre-near work.

**Fig. 3:**
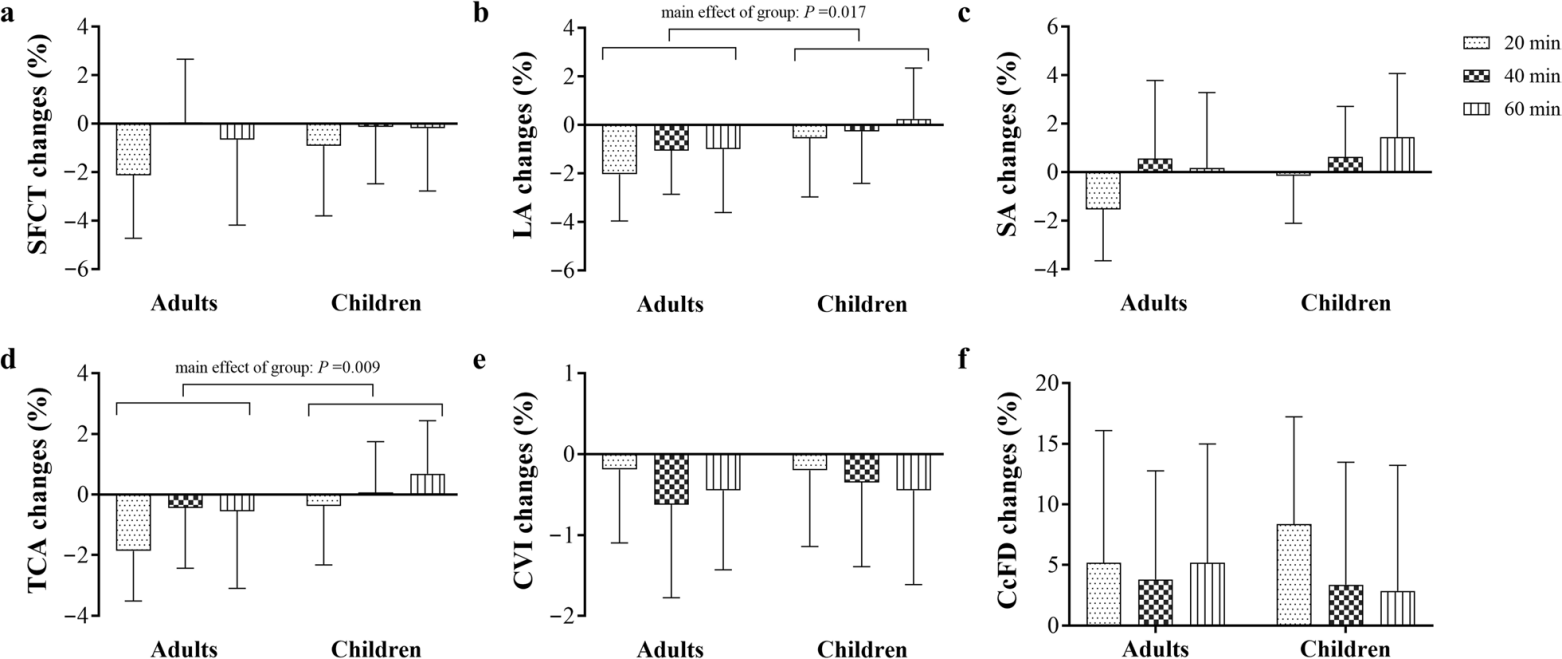
Percentage changes of choroidal metrics during near work. Percentage changes of (**a**) subfoveal choroidal thickness (SFCT), (**b**) luminal area (LA), (**c**) stromal area (SA), (**d**) total choroidal area (TCA), (**e**) choroidal vascularity index (CVI) and (**f**) choriocapillaris flow deficits (CcFD) during near work for children and adults

Across all participants, the magnitude of SFCT, LA, SA and TCA changes varied significantly after 20, 40 and 60 min of near work (main effect: SFCT: *P* = 0.004; LA: *P* = 0.009; SA: *P* < 0.001; TCA: *P* < 0.001, Fig. 3, Additional file 5), with the greatest reduction seen at 20 min. Adults exhibited a greater reduction of 0.86% in LA changes than that observed in children (main effect: *P* = 0.017), as well as a greater reduction of 0.82% in TCA changes (main effect: *P* = 0.009). The changes in other choroidal metrics were not significantly different between the two age groups (all *P* > 0.05). The interactions between time and age group for all choroidal metrics were not significant (all *P* > 0.05).

## Discussion

In this study, we studied the choroidal vasculature by analyzing the LA, SA and TCA, which indicated the vascularity of medium- and large-sized vessels layer, and CcFD, which indicated the choriocapillaris perfusion in response to near work. We identified that the temporal characteristics and magnitude of changes in different choroidal layers were different in children and adults. In adults, 20 min of near work decreased the SFCT, submacular LA, SA and TCA, while 40 min of near work decreased the LA and CVI and increased CcFD. After 60 min of near work, CVI was still decreased and CcFD was still increased. On the other hand, in children, 20 min of near work induced an increase in CcFD, while 60 min of near work increased the SA and TCA and decreased the CVI. The reduction in LA and TCA in children was lower than in adults.

### Effect and temporal properties of near work on choroid

Studies on myopia and near work have traditionally aimed at the choroidal thickness changes to an accommodative stimulus [20, 23, 33]. Intensity and duration of near work can influence the magnitude of choroidal thickness changes. The choroid thinned significantly with a higher degree of accommodative stimulus. Choroidal thickness decreased with a 6.00 D stimulus at 10 min and 30 min [20, 23, 33], while no significant changes were observed with a 3.00 D stimulus during those periods [23–25]. A similar phenomenon was observed in animals reared under different visual environments with exposures to near, middle and long viewing distances [19]. Besides, the time course also influenced the choroidal thickness changes. In our study, choroidal thickness in adults showed a significant decrease in early stage (20 min) and then this reduction weakened. Similarly, Woodman et al. found choroidal thinning with − 4.00 D accommodation demand in early stages (5 to 10 min), then saw the reduction weakened progressively [22]. These changes in choroidal thickness indicated temporal effect of sustained accommodation was not simply a linear accumulation.

The studies on choroidal vascularity and blood flow signal in response to visual stimuli are limited. Pan et al. and Liang et al. found a significant increase in CcFD and a significant decrease in LA and CVI in young adults after 40 min of near work [24, 25], which was consistent with our results. Chang et al. showed that LA, SA and TCA significantly decreased in myopic children with additional − 3.00 D lenses on top of the fully corrected prescription lenses after 30 min of near work (about 6.00 D stimulus) [20]. However, in our study, the changes in LA, SA and TCA in children after 20 and 40 min of near work did not reach statistical significance. This may be because our 3.00 D stimulus was not adequate to induce similar magnitudes of changes as the 6.00 D stimulus. Furthermore, no significant changes in choriocapillaris perfusion were found in their study, while a significant reduction was observed in children in our study after 20 min of near work. This difference may be attributed to the different raster scan protocol that covers an area of 3 mm × 3 mm in our study as opposed to 6 mm × 6 mm used in their study. Overall, our data suggest that changes in medium- and large-sized vascular layers and the choriocapillaris layer responding to the near work might not be synchronous in the temporal characteristics.

### The difference in choroidal response to near work

Our results showed that the choroidal responses to near work in children and adults were different in temporal pattern and magnitude. The initial response to near work was observed in choriocapillaris in children, whereas it was mainly in the medium- and large-sized vessel layers in adults. As the choroid lies behind the RPE layer and cannot respond readily to retinal metabolic or neurovascular signals, retinal activity-dependent regulation of choroidal blood flow is mediated by retinal input to central autonomic circuits [17, 34]. The changes in medium- and large-sized vessel layers may be due to neuronal control since parasympathetic, sympathetic, and sensory fibers and their terminals tend to be localized to the walls of the arteries and veins of the choroid, not the choriocapillaris layer [34]. Intrinsic choroidal neurons (ICNs) probably also play a role in choroidal blood flow as putative targets innervated by ICNs are arteries and nonvascular smooth muscle fibers [35, 36]. However, because sympathetic, parasympathetic and sensory fibers end at the subcapillary level, instead of the choriocapillaris level, the changes in choriocapillaris perfusion may be regulated indirectly by the incoming/outgoing choroidal blood flow [37].

The difference in choroidal responses between children and adults may be ascribed to the effect of development, including choroidal vascularity, innervation and non-vascular smooth muscle. Though relevant studies on choroid in children and young adults are scarce, some studies highlighted the difference in choroidal morphometrics [38], choroidal perfusion [29, 39, 40], endothelial cells [41–43], choroidal nerve fibers [44–46], and non-vascular smooth muscle in aging [47]. In summary, it is evident from our data that the choroidal responses vary among the two age groups. Further studies are warranted to narrow down the factors mediating these differences.

### Limitations

The limitations of this study include lack of data on choroidal metrics at time points earlier than 20 min. Shorter durations of near work and continuous monitoring would provide a better understanding of choroidal changes. Another possible limitation could be that the study was performed in myopic eyes rather than emmetropic eyes. It was reported that the choroidal thickness changes in response to accommodative stimuli vary between myopes and emmetropes, with little choroidal responses to accommodation in emmetropes [20, 22]. Whether the difference we found exists in emmetropic eyes warrants further studies. In this study, AL varied greatly, reflecting the difference in ocular structure between children and adults. This difference may probably reflect on their choroidal vasculature as the choroid thinning is accompanied by ocular elongation [48, 49]. Therefore, we adjusted for AL and calculated the percentage changes of choroidal metrics to reduce the influences. Meanwhile, we acknowledge that non-cycloplegic subjective refraction could result in a more myopic refractive error than a cycloplegic autorefraction [50]. We employed subjective refraction mainly to obtain a full correction prescription for the reading task, hence this method was preferred over a cycloplegic refraction [51, 52].

## Conclusions

Choroidal vascularity and choriocapillaris perfusion responded differently to near work in children and young adults. The responses to near work were not identical in temporal characteristics and magnitude of changes. The initial response to near work was observed in the choriocapillaris in children, whereas its was observed in the medium- and large-sized vessels in adults. Overall, this study provides new insights on varying responses of different choroidal components to near work in different age groups.

### Supplementary Information

**Additional file 1.** Illustration of the time course of the experiments.

**Additional file 2.** Bland-Altman plots for differences of choroidal metrics between the two serials of images captured consecutively.

**Additional file 3.** Choroidal metrics of pre- and post-near work in adults (n=30).

**Additional file 4.** Choroidal metrics of pre- and post-near work in children (n=30).

**Additional file 5.** Percentage changes of choroidal metrics during near work.

## Data Availability

Data are available upon reasonable request.

## Abbreviations

AL: Axial length
BCVA: Best corrected visual acuity
BM: Bruch’s membrane
CcFD: Choriocapillaris flow deficit
CVI: Choroidal vascularity index
ICC: Intraclass correlation coefficient
IOP: Intraocular pressure
LA: Luminal area
OCT: Optical coherence tomography
OCTA: Optical coherence tomography angiography
RPE: Retinal pigment epithelium
SA: Stromal area
SER: Spherical equivalent refraction
SFCT: Subfoveal choroidal thickness
SS-OCT: Swept-source optical coherence tomography
TCA: Total choroidal area
