# Supplementary figures and images for: Differences in choroidal responses to near work between myopic children and young adults
Source: Eye Vis (Lond). 2024 Apr 2;11:12. doi: 10.1186/s40662-024-00382-5 (PMC10986059; doi:10.1186/s40662-024-00382-5)

**Additional file 1. Illustration of the time course of the experiments.**

**
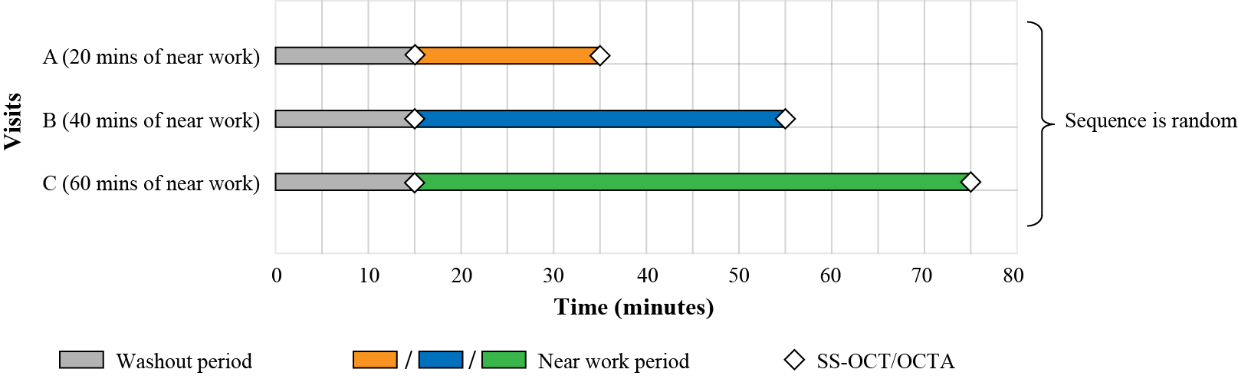
**

Supplement: Supplementary file 1 — Additional file 1. Illustration of the time course of the experiments. [file 40662_2024_382_MOESM1_ESM.docx]
