# Supplementary material for: Differences in choroidal responses to near work between myopic children and young adults
Source: Eye Vis (Lond). 2024 Apr 2;11:12. doi: 10.1186/s40662-024-00382-5 (PMC10986059; doi:10.1186/s40662-024-00382-5)

**Additional file 2. Bland-Altman plots for differences of choroidal metrics between the two serials of images captured** **consecutively.** **a** Subfoveal choroidal thickness (SFCT); **b** Luminal area (LA); **c** Stromal area (SA); **d** Total choroidal area (TCA); **e** Choroidal vascularity index (CVI); **f** Choriocapillaris flow deficit (CcFD). The intraclass correlation coefficients (ICCs) of SFCT, LA, SA, TCA, CVI and CcFD varied from 0.962 to 0.998 for intra-capture repeatability.


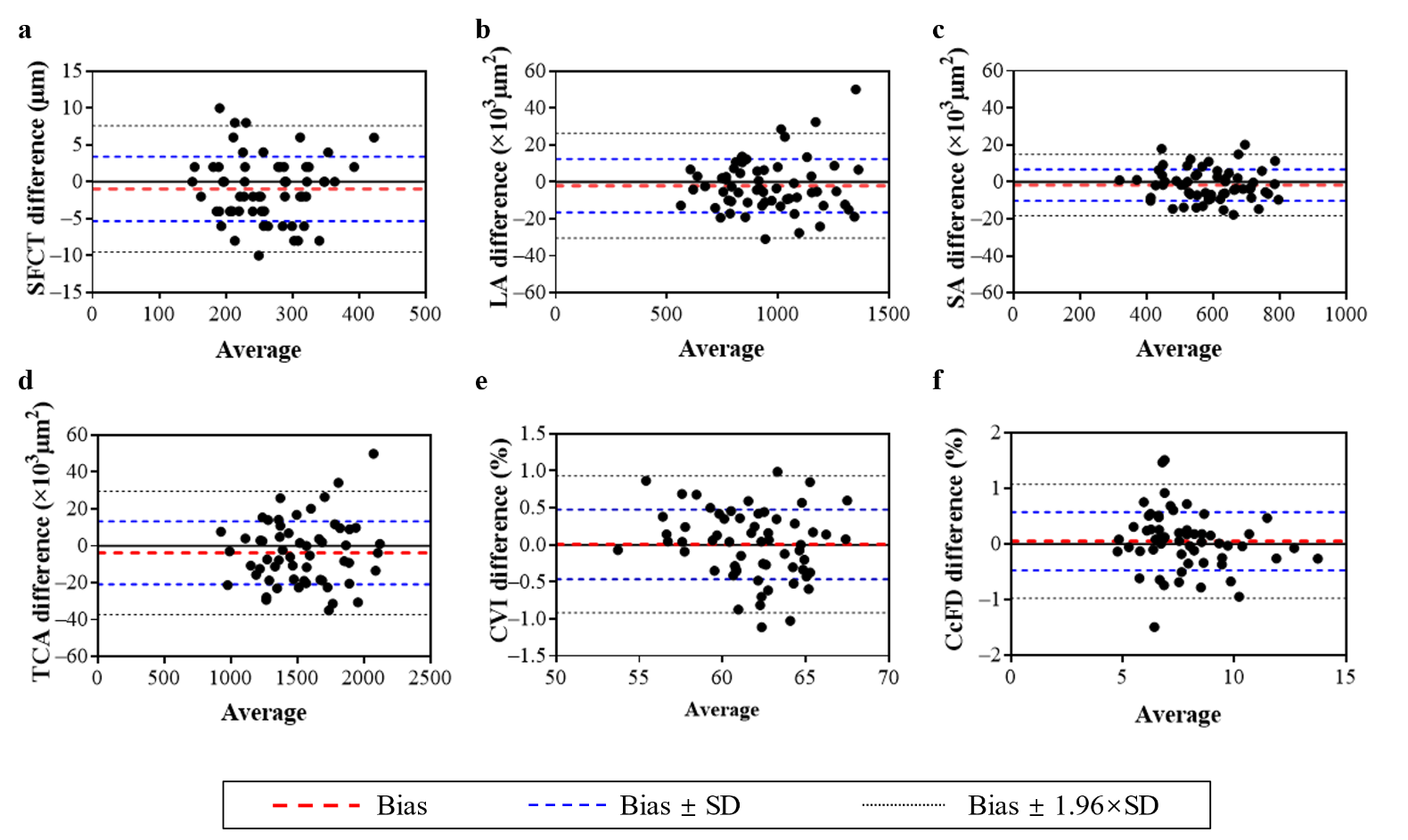

Supplement: Supplementary file 2 — Additional file 2. Bland-Altman plots for differences of choroidal metrics between the two serials of images captured consecutively. [file 40662_2024_382_MOESM2_ESM.docx]
