# Supplementary material for: Differences in choroidal responses to near work between myopic children and young adults
Source: Eye Vis (Lond). 2024 Apr 2;11:12. doi: 10.1186/s40662-024-00382-5 (PMC10986059; doi:10.1186/s40662-024-00382-5)
Supplement: Supplementary file 3 — Additional file 3. Choroidal metrics of pre- and post-near work in adults (n=30). [file 40662_2024_382_MOESM3_ESM.docx]

**Additional file 3.** Choroidal metrics of pre- and post-near work in adults (n=30).

| **Parameter** | **Time** | **Pre-near work** | **Post-near work** | **Changes** | ***P* value*** |
| --- | --- | --- | --- | --- | --- |
| SFCT (μm) |  |  |  |  |  |
|  | 20mins | 268.0±67.3 | 262.8±69.1 | −5.1±6.5 | <0.001 |
|  | 40mins | 264.2±67.3 | 264.2±67.4 | 0.0±6.4 | >0.999 |
|  | 60mins | 267.2±68.5 | 265.9±70.4 | −1.3±8.9 | 0.444 |
|  | ***P* value**# | 0.069 |  |  |  |
| LA (×10^3^μm^2^) |  |  |  |  |  |
|  | 20mins | 990.5±229.2 | 971.3±230.8 | −19.2±18.6 | <0.001 |
|  | 40mins | 985.0±227.8 | 975.7±231.7 | −9.4±18.3 | 0.009 |
|  | 60mins | 988.6±228.1 | 981.1±236.8 | −7.5±24.8 | 0.108 |
|  | ***P* value**# | 0.477 |  |  |  |
| SA (×10^3^μm^2^) |  |  |  |  |  |
|  | 20mins | 591.8±112.1 | 583.6±116.5 | −8.2±12.6 | 0.001 |
|  | 40mins | 582.8±110.4 | 586.2±113.8 | 3.4±18.5 | 0.318 |
|  | 60mins | 588.3±116.3 | 588.9±116.0 | 0.6±17.3 | 0.847 |
|  | ***P* value**# | 0.058 |  |  |  |
| TCA (×10^3^μm^2^) |  |  |  |  |  |
|  | 20mins | 1582.3±330.7 | 1554.9±337.0 | −27.4±24.9 | <0.001 |
|  | 40mins | 1567.9±327.5 | 1561.9±333.7 | −5.9±31.3 | 0.306 |
|  | 60mins | 1576.9±334.4 | 1570.0±343.6 | −6.9±39.2 | 0.344 |
|  | ***P* value**# | 0.136 |  |  |  |
| CVI (%) |  |  |  |  |  |
|  | 20mins | 62.34±3.08 | 62.22±3.06 | −0.12±0.56 | 0.242 |
|  | 40mins | 62.58±3.09 | 62.19±3.24 | −0.39±0.70 | 0.005 |
|  | 60mins | 62.48±2.84 | 62.21±3.02 | −0.28±0.59 | 0.015 |
|  | ***P* value**# | 0.156 |  |  |  |
| CcFD (%) |  |  |  |  |  |
|  | 20mins | 8.42±2.46 | 8.74±2.37 | 0.33±0.93 | 0.065 |
|  | 40mins | 8.36±2.12 | 8.66±2.29 | 0.30±0.78 | 0.045 |
|  | 60mins | 8.37±2.18 | 8.74±2.16 | 0.37±0.75 | 0.012 |
|  | ***P* value**# | 0.911 |  |  |  |

SFCT = subfoveal choroidal thickness; LA = luminal area; SA = stromal area; TCA = total choroidal area; CVI = choroidal vascularity index; CcFD = choriocapillaris flow deficits.

*P* values were determined by repeated measures ANOVA with Bonferroni post-hoc test. Time (20 mins, 40 mins and 60 mins) and near work (pre-near work and post-near work) were within-subject factors.

* Comparison between post-near work with pre-near work choroidal metrics.

# Comparison among the three sessions for the pre-near work choroidal metrics.
