# Supplementary material for: Differences in choroidal responses to near work between myopic children and young adults
Source: Eye Vis (Lond). 2024 Apr 2;11:12. doi: 10.1186/s40662-024-00382-5 (PMC10986059; doi:10.1186/s40662-024-00382-5)
Supplement: Supplementary file 4 — Additional file 4. Choroidal metrics of pre- and post-near work in children (n=30). [file 40662_2024_382_MOESM4_ESM.docx]

**Additional file 4.** Choroidal metrics of pre- and post-near work in children (n=30).

| **Parameter** | **Time** | **Pre-near work** | **Post-near work** | **Changes** | ***P* value*** |
| --- | --- | --- | --- | --- | --- |
| SFCT (μm) |  |  |  |  |  |
|  | 20mins | 255.1±52.8 | 252.5±51.7 | −2.5±8.4 | 0.109 |
|  | 40mins | 255.5±54.6 | 254.7±53.2 | −0.7±5.9 | 0.500 |
|  | 60mins | 256.4±51.4 | 255.9±51.9 | −0.5±6.2 | 0.685 |
|  | ***P* value#** | 0.694 |  |  |  |
| LA (×10^3^μm^2^) |  |  |  |  |  |
|  | 20mins | 917.0±164.3 | 912.1±165.9 | −4.9±24.4 | 0.279 |
|  | 40mins | 920.1±173.6 | 916.5±168.7 | −3.6±20.3 | 0.346 |
|  | 60mins | 920.9±165.7 | 923.3±168.0 | 2.4±20.0 | 0.509 |
|  | ***P* value#** | 0.727 |  |  |  |
| SA (×10^3^μm^2^) |  |  |  |  |  |
|  | 20mins | 587.4±114.7 | 586.5±114.9 | −0.9±12.9 | 0.704 |
|  | 40mins | 586.0±114.5 | 589.7±116.5 | 3.7±12.4 | 0.112 |
|  | 60mins | 587.4±115.6 | 594.6±112.7 | 7.2±13.0 | 0.005 |
|  | ***P* value#** | 0.887 |  |  |  |
| TCA (×10^3^μm^2^) |  |  |  |  |  |
|  | 20mins | 1504.4±267.1 | 1498.5±266.7 | −5.8±32.9 | 0.340 |
|  | 40mins | 1506.1±275.8 | 1506.2±273.1 | 0.1±26.1 | 0.977 |
|  | 60mins | 1508.3±269.5 | 1518.0±269.4 | 9.7±25.3 | 0.046 |
|  | ***P* value#** | 0.872 |  |  |  |
| CVI (%) |  |  |  |  |  |
|  | 20mins | 61.03±2.70 | 60.91±2.90 | −0.12±0.57 | 0.276 |
|  | 40mins | 61.10±2.76 | 60.89±2.74 | −0.22±0.63 | 0.069 |
|  | 60mins | 61.12±2.71 | 60.84±2.61 | −0.28±0.72 | 0.040 |
|  | ***P* value#** | 0.775 |  |  |  |
| CcFD (%) |  |  |  |  |  |
|  | 20mins | 6.82±1.32 | 7.37±1.52 | 0.55±0.64 | <0.001 |
|  | 40mins | 7.11±1.47 | 7.30±1.48 | 0.19±0.67 | 0.134 |
|  | 60mins | 7.05±1.38 | 7.22±1.54 | 0.17±0.74 | 0.212 |
|  | ***P* value#** | 0.165 |  |  |  |

SFCT = subfoveal choroidal thickness; LA = luminal area; SA = stromal area; TCA = total choroidal area; CVI = choroidal vascularity index; CcFD = choriocapillaris flow deficits.

*P* values were determined by repeated measures ANOVA with Bonferroni post-hoc test. Time (20 mins, 40 mins and 60 mins) and near work (pre-near work and post-near work) were within-subject factors.

* Comparison between post-near work with pre-near work choroidal metrics.

# Comparison among the three sessions for the pre-near work choroidal metrics.
