# Supplementary material for: Differences in choroidal responses to near work between myopic children and young adults
Source: Eye Vis (Lond). 2024 Apr 2;11:12. doi: 10.1186/s40662-024-00382-5 (PMC10986059; doi:10.1186/s40662-024-00382-5)
Supplement: Supplementary file 5 — Additional file 5. Percentage changes of choroidal metrics during near work. [file 40662_2024_382_MOESM5_ESM.docx]

**Additional file 5.** Percentage changes of choroidal metrics during near work.

| **Parameter** | **Time** | **Adults** | **Children** | **Differences**  **(Adults−Children)*** | ***P* value*** | | | |
| --- | --- | --- | --- | --- | --- | --- | --- | --- |
|  |  |  |  |  | **Time** | **Group** | **Time by Group** | **AL** |
| SFCT (%) |  |  |  |  | 0.004 | 0.602 | 0.325 | 0.027 |
|  | 20 mins | −2.13±2.59 | −0.92±2.88 | −0.95 |  |  |  |  |
|  | 40 mins | 0.02±2.64 | −0.12±2.36 | 0.42 |  |  |  |  |
|  | 60 mins | −0.66±3.53 | −0.19±2.59 | −0.21 |  |  |  |  |
|  | Means* | −0.79 | −0.54 | −0.25 |  |  |  |  |
| LA (%) |  |  |  |  | 0.009 | 0.017 | 0.594 | <0.001 |
|  | 20 mins | −2.04±1.92 | −0.55±2.42 | −1.17 |  |  |  |  |
|  | 40 mins | −1.06±1.80 | −0.27±2.14 | −0.47 |  |  |  |  |
|  | 60 mins | −1.00±2.62 | 0.24±2.11 | −0.92 |  |  |  |  |
|  | Means* | −1.21 | −0.35 | −0.86 |  |  |  |  |
| SA (%) |  |  |  |  | <0.001 | 0.064 | 0.218 | 0.065 |
|  | 20 mins | −1.54±2.12 | −0.15±1.95 | −1.22 |  |  |  |  |
|  | 40 mins | 0.56±3.22 | 0.63±2.08 | 0.10 |  |  |  |  |
|  | 60 mins | 0.16±3.12 | 1.44±2.62 | −1.12 |  |  |  |  |
|  | Means* | −0.19 | 0.56 | −0.75 |  |  |  |  |
| TCA (%) |  |  |  |  | <0.001 | 0.009 | 0.296 | <0.001 |
|  | 20 mins | −1.86±1.65 | −0.38±1.94 | −1.22 |  |  |  |  |
|  | 40 mins | −0.45±1.98 | 0.07±1.67 | −0.25 |  |  |  |  |
|  | 60 mins | −0.55±2.54 | 0.68±1.75 | −0.98 |  |  |  |  |
|  | Means* | −0.82 | −0.01 | −0.82 |  |  |  |  |
| CVI (%) |  |  |  |  | 0.125 | 0.871 | 0.603 | 0.060 |
|  | 20 mins | −0.19±0.90 | −0.20±0.94 | 0.07 |  |  |  |  |
|  | 40 mins | −0.62±1.15 | −0.35±1.04 | −0.21 |  |  |  |  |
|  | 60 mins | −0.45±0.98 | −0.45±1.17 | 0.06 |  |  |  |  |
|  | Means* | −0.39 | −0.36 | −0.03 |  |  |  |  |
| CcFD (%) |  |  |  |  | 0.069 | 0.886 | 0.200 | 0.651 |
|  | 20 mins | 5.19±10.89 | 8.36±8.84 | −3.35 |  |  |  |  |
|  | 40 mins | 3.81±8.96 | 3.37±10.10 | 0.27 |  |  |  |  |
|  | 60 mins | 5.19±9.80 | 2.58±10.64 | 2.43 |  |  |  |  |
|  | Means* | 4.64 | 4.86 | −0.22 |  |  |  |  |

SFCT = subfoveal choroidal thickness; LA = luminal area; SA = stromal area; TCA = total choroidal area; CVI = choroidal vascularity index; CcFD = choriocapillaris flow deficits; AL = axial length.

*Estimated marginal means, differences and *P* values were determined by generalized estimating equations (GEE), adjusted for axial length.
